# So, what is chiropractic? Summary and reflections on a series of papers in Chiropractic and Manual Therapies

**DOI:** 10.1186/s12998-019-0295-2

**Published:** 2020-01-30

**Authors:** Jan Hartvigsen, Simon D. French

**Affiliations:** 1 Department of Sports Science and Clinical Biomechanics, University of Southern Denmark, Campusvej 55, 5230 Odense M, Denmark; 2 Nordic Institute of Chiropractic, Campusvej 55, 5230 Odense M, Denmark; 3 Department of Chiropractic, Macquarie University, Sydney, NSW 2109 Australia

**Keywords:** Chiropractic, Education, Disability, X-ray, Debate

## Abstract

This commentary brings the 2017–2019 thematic series *What is Chiropractic?* to a close. The 18 papers published in the series contribute to a better understanding of what chiropractic is, where chiropractors practice and function, who seeks their care, what chiropractors do, and how they interact with other healthcare professionals. Several papers in the series highlighted deeply rooted disagreements within chiropractic about fundamental issues pertaining to ideology, acceptance of scientific evidence as the basis for clinical practice and the future of chiropractic. If the chiropractic profession is to remain relevant in today’s evidence-based healthcare environment, there is an urgent for the profession to undertake further research to describe what chiropractic is, what chiropractors do, and provide evidence for the value of these activities to patients and healthcare decision makers.

## Background

In November of 2017, *Chiropractic and Manual Therapies* issued a call for papers under the common theme “*What is Chiropractic?”.* The motivation for the thematic series was to help define chiropractic better to stakeholders inside and outside of the profession. The need for the thematic series was a perceived confusion about what chiropractic is, what chiropractors do, and how they contribute to the health and well-being of patients [1] in spite of official definitions such as by the World Federation of Chiropractic: *Chiropractic is a health profession concerned with the diagnosis, treatment and prevention of mechanical disorders of the musculoskeletal system, and the effects of these disorders on the function of the nervous system and general health. There is an emphasis on manual treatments including spinal adjustment and other joint and soft-tissue manipulation* [2]. Two years later, 18 papers have been published in the series, and it is time to take stock and examine what we have learned through this initiative.

## Main text

We have grouped the published papers in the thematic series under three headings and summarize them below. An overview of aims and conclusions of papers in this series is provided in Table 1.

**Table 1:** Aims and conclusions of papers published in the series What is chiropractic?

First author; year; title; study design	Aim of paper	Conclusion
Papers describing the chiropractic profession and chiropractic practice		
Beliveau et al. 2017. *The chiropractic profession: a scoping review of utilization rates, reasons for seeking care, patient profiles, and care provided.* Scoping review	To document the current state of knowledge on the: 1) utilization of chiropractic services; 2) reasons for attending chiropractic care; 3) demographic and health profiles of chiropractic patients; and 4) types of chiropractic assessment and treatment provided worldwide.	Across different countries and regions, the average 12- month utilization rate of chiropractic services was 9.1% with little change between 1980 and 2015. Musculoskeletal conditions, such as back and neck pain, were the predominant reason for people attending chiropractic care. Typically, chiropractic patients were female, aged 43.4 years, and employed. Four out of five patients who consulted a chiropractor received spinal manipulation; however, chiropractors also commonly provided other treatments including soft-tissue therapy and formal patient education.
Stochkendahl et al. 2018. *Can chiropractors contribute to work disability prevention through sickness absence management for musculoskeletal disorders?* Comparative qualitative case study	To describe the experiences of chiropractors engaging in sickness absence management (SAM) and to compare and contrast chiropractors’ integration of SAM in their model of care in a context with legislated sickness certification rights (Norway) and in two contexts without sickness certification rights (Sweden and Denmark).	Chiropractors with patient management expertise can fulfil a key role in SAM and by extension work disability prevention when these practices are legislatively supported. In cases where these practices occur informally, however, practitioners face systemic-related issues and professional self-image challenges that tend to hamper them in fulfilling a more integrated role as providers of WDP practices.
Salsbury et al. 2018. *Be good, communicate, and collaborate: a qualitative analysis of stakeholder perspectives on adding a chiropractor to the multidisciplinary rehabilitation team.* Qualitative analysis based on personal and focus group interviews	To explore stakeholder perceptions of the qualities preferred in a chiropractor from the perspectives of patients, families, and interdisciplinary team members affiliated with this rehabilitation setting.	Study participants supported the addition of a chiropractor to the multidisciplinary team who practiced in a safe, evidence-based, patient-centered manner. Interprofessional skills that enhanced teamwork, intrapersonal qualities to support patients’ emotional journeys through the rehabilitation process, and an organizational perspective that amplified the mission of the institution also were desired.
Jenkins et al. 2018. *Current evidence for spinal X-ray use in the chiropractic profession.* Narrative review	To review 1) the current use of spinal X-ray imaging within chiropractic clinical practice; 2) the evidence for potential reasons for obtaining spinal X-rays within chiropractic; 3) the evidence of possible risks or limitations associated with the use of spinal X-rays; and 4) guidelines for the appropriate use of imaging in chiropractic clinical practice.	The use of spinal X-rays in chiropractic has been controversial, with benefits for the use of routine spinal X-rays being proposed by some elements of the profession. However, evidence of these postulated benefits is limited or non-existent. There is strong evidence to demonstrate potential harms associated with spinal X-rays including increased ionizing radiation exposure, overdiagnosis, subsequent low-value investigation and treatment procedures, and increased unnecessary costs. Therefore, in the vast majority of cases who present to chiropractors, the potential benefit from spinal X-rays does not outweigh the potential harms.
Hansen et al. 2018 *Work-related acute physical injuries, chronic overuse complaints, and the psychosocial work environment in Danish primary care chiropractic practice - a cross-sectional study.* Cross-sectional study	To describe work-related physical acute injuries and overuse complaints as well as psychosocial stress among chiropractors in primary care chiropractic practice in Denmark during the previous year.	Danish practicing chiropractors commonly reported physical work-related acute injuries or overuse complaints. Overuse complaints are significantly more common in women and occur primarily in the low back, wrist, thumb, and shoulder. Chiropractors with less than five years in practice report more overuse complaints than chiropractors with more than five years in practice. Chiropractors in Denmark generally have a good psychosocial work environment.
Innes et al. 2018 *Comparing the old to the new: A comparison of similarities and differences of the accreditation standards of the chiropractic council on education-international from 2010 to 2016.* Review of published accreditation standards	1. to compare the Council on Chiropractic Education International 2016 Accreditation Standards with their previous 2010 Accreditation Standards, including the way they were developed, and; 2. to explore similarities and differences of prescribed recommendations to identify any changes to procedures, concepts, and emphases; 3. to comment on whether these changes are likely to be for the better or the worse.	Some positive changes have taken place, such as having bravely moved towards the musculoskeletal model, but on the negative side, the requirement to produce graduates skilled at dealing with scientific texts has been removed. A more robust development approach including better transparency is needed before implementation of CCE standards and evidence-based concepts should be integrated in the programs. The CCE-International should consider the creation of a recognition of excellence in educational programs and not merely propose minimal standards.
Funk et al. 2018 *The prevalence of the term subluxation in chiropractic degree program curricula throughout the world.* Review of websites	To determine the prevalence of the term subluxation in the course titles or descriptions in all chiropractic training programs around the world for which information could be found.	The term subluxation was found in all but two US chiropractic course catalogues. The term was mentioned over eight times more frequently in US than non-US course catalogues. Similarly, subluxation was found greater than nine times more frequently in US course descriptions than in non-US descriptions.
Stochkendahl et al. 2019. *The chiropractic workforce: a global review.* Cross-sectional survey	To describe the chiropractic workforce worldwide in terms of the number of chiropractors, education, access, reimbursement schemes, scope of practice, and legal rights.	The [chiropractic] profession is represented in 90 countries, but the distribution, chiropractic educational institutions, and governing legislations and regulations largely favour high-income countries. There is a large under-representation in low- and middle-income countries in terms of provision of services, education and legislative and regulatory frameworks, and the available data from these countries are limited.
Stochkendahl et al. 2019. *Managing sickness absence of patients with musculoskeletal pain - a cross-sectional survey of Scandinavian chiropractors.* Cross-sectional survey	To 1) determine the prevalence of musculoskeletal practitioners’ key practice behaviors, and perceptions and beliefs about SAM using Scandinavian chiropractors; 2) to determine what characteristics of the practitioners, practice behaviors, perceptions and beliefs, and country were associated with two different practice behaviors.	Whilst not always engaged in sickness absence management (SAM) with regards to musculoskeletal pain, chiropractors favor a ‘return-to-work’ rather than a ‘stay-at-home’ approach. Norwegian chiropractors who have the right to prescribe sick leave consistently report more positive perceptions and beliefs towards SAM and a greater level of involvement with the process compared with the Danish and Swedish chiropractors who do not have these rights. Several practice behaviors and perceptions and beliefs are associated with these outcomes; however, system or organizational barriers are linked to clinician non-engagement.
Mior et al. 2019. *Chiropractic services in the active duty military setting.* Scoping review	To describe 1) access of chiropractic services; 2) chiropractic scope of practice, e.g. procedures, processes, and actions; 3) service model and location; and 4) type of condition treated, duration, and outcomes of treatment provided to active duty military members.	The majority of the articles emanated from the US and were cross-sectional in nature. Two recent RCTs provide evidence of comparative effectiveness of adding chiropractic care to usual medical care. Despite the reported use of chiropractic services in Australia, Canada, and the US, there is little available published evidence related to the nature, use, and outcomes of chiropractic care in active duty military.
Wirth et al. 2019 *An observational study on trajectories and outcomes of chronic low back pain patients referred from a spine surgery division for chiropractic treatment.* Longitudinal cohort study	To describe the trajectories and outcomes of patients with chronic LBP referred from the spine surgery division to the chiropractic teaching clinic.	Chronic LBP patients with long-lasting pain, reduced general health and high bio-psycho-social impairment, referred from spine surgery to a chiropractic teaching clinic, benefit from being co-managed by surgeons and chiropractors. Present pain intensity and Bournemouth Questionnaire score for bio-psycho-social impairment diminished by about 25% within the first 12 months after the start of chiropractic treatment.
Axen et al. 2019 *Chiropractic maintenance care – what’s new?* Systematic review	1. To define the concept of Maintenance Care and the indications for its use; 2. To describe chiropractors’ belief in Maintenance Care and patients’ acceptance of it; 3. To establish the prevalence with which chiropractors use Maintenance Care and possible characteristics of the chiropractors associated with its use; 4. To determine its efficacy and cost-effectiveness for various types of conditions.	Back pain is a chronic disease for most, with episodes at short or long intervals. A preventive approach [to back pain] such as Maintenance Care makes sense. It is still not known if it ‘works’ because of the treatment given or because of the clinical encounter, or how these two components interact.
Gislason et al. 2019. *The shape of chiropractic in Europe: a cross sectional survey of chiropractor’s beliefs and practice.* Cross-sectional survey	To repeat a Canadian study within Europe aiming at categorizing beliefs or potential association with unorthodox practice.	Around one fifth of European chiropractors completing this survey identified themselves with an a priori defined unorthodox description of chiropractic care, i.e. “*treating vertebral subluxations as an encumbrance to the expression of health”*. The data also showed a number of key predictive practice behaviors statistically associated. with this unorthodox group, including higher use of x-rays, higher patient visits, absence of differential diagnostic approaches and less favorable views regarding the benefits of vaccination.
Papers describing new initiatives and developments in the chiropractic profession		
Adams et al. 2018. *Leadership and capacity building in international chiropractic research: introducing the chiropractic academy for research leadership (CARL).* Commentary	To describe the aims, core principles, methodology and evolution of the Chiropractic Academy of Research Leadership (CARL).	The long-term aim of CARL is to develop essential leadership skills and experiences to take on senior CARL mentorship appointments and help secure the successful mentorship of a subsequent early-career researcher cohort of Fellows.
Côté et al. 2019. *The development of a global chiropractic rehabilitation competency framework by the World Federation of Chiropractic.* Commentary	To present a chiropractic rehabilitation competency framework developed by the World Federation of Chiropractic Disability and Rehabilitation Committee.	Presents a chiropractic rehabilitation framework consisting of three domains: basic concepts of rehabilitation and disability; legal, regulatory and ethical components; and, rehabilitation management of disability and other health conditions.
Discussion and debate papers		
Coulter et al. 2019. *The research crisis in American institutions of complementary and integrative health: one proposed solution for chiropractic profession.* Commentary	To discuss the overall crisis in research in complementary and alternative medicine and propose solutions with a special focus on chiropractic.	If the institutions pool their limited talent and resources, they might be able to compete, but history has shown that the American chiropractic institutions have not yet been able to do that. However, perhaps through a mediator it might be achievable. We are not suggesting this proposal will solve all the problems and we recognize that outside of the US the situation is different. But within the US it seems to us that without some type of response the situation will get worse not better. The RAND Health program has signed onto this proposal and we are currently approaching foundations to fund it.
Leboeuf-Yde et al. 2019. *Chiropractic, one big unhappy family: better together or apart?* Commentary	To examine the chiropractic profession from the perspective of an unhappy marriage by defining key elements in happy and unhappy marriages and by identifying factors that may determine why couples stay together or spilt up.	Chiropractors and chiropractic leaders, regardless of values and persuasion, need to pause and consider, if they are able to live and develop as they would like to in this century-old unhappy marriage.
Strahinjevich and Simpson 2018. *The schism in chiropractic through the eyes of a 1st year chiropractic student.* Commentary	To review the historical origins of the schism in chiropractic and examine the influence the schism has had on the profession and to discuss a possible strategy whereby this schism can be healed.	The schism as it is known has been identified as a division between those who adhere to the dogma of Palmerian ideology and those who embrace scientific advancement. Also considered were some of the reasons, largely external to the profession for the perpetuation of the divide.

### Papers describing the chiropractic profession and chiropractic practice

The overall global view of the chiropractic profession was provided in two articles. Stochkendahl et al. provided a global view of the chiropractic workforce and concluded that chiropractors are present in 90 of 193 United Nations member nations with the highest prevalence in high-income countries [3]. Although chiropractors are legally recognized in 75% of those countries, and chiropractic treatment is fully or partially subsidized by national healthcare systems in around 50%, in some countries chiropractic does not fall under any law or may even be illegal [3]. Beliveau et al. reviewed 52 studies dealing with utilization of chiropractic and found that, generally, utilization was highly variable and population dependent, with no clear change in utilization of chiropractors globally between 1980 and 2015 [4]. They found that the typical chiropractic patient is seeking care for a musculoskeletal problem, most commonly low back pain (50%) and neck pain (23%), is female, middle-aged and employed [4]. Although there is some variation in practice styles, most people seeking care from chiropractors will receive spinal manipulation with around one third also receiving patient education, exercises and soft-tissue therapy [4].

There is an increasing body of literature describing chiropractors in interprofessional collaborations. Mior et al. reviewed literature dealing with chiropractors functioning in the health services of armed forces mainly from the US, Canada and Australia [5]. Stochkendahl et al. found that in the Scandinavian context, chiropractors can fulfil a key role in management of sickness absence and in work disability prevention both informally and as part of system-supported pathways [6, 7]. Another example of chiropractors functioning in interprofessional collaboration was provided by Wirth et al. who reported on outcomes and trajectories of patients referred from a surgical department to chiropractic care [8]. In these settings, chiropractors frequently work alongside or in collaboration with other providers, and Salsbury et al. reported, based on a qualitative stakeholder analysis, that in order to be successful in multidisciplinary teams, chiropractors should be good clinicians, practice according to evidence, be communicative and be supportive of patient’s emotional journeys during care and rehabilitation [9].

Four articles dealt with different elements of chiropractic practice. Axén et al. systematically reviewed evidence pertaining to chiropractic maintenance care and concluded that significant advances had been made in recent decades regarding what maintenance care is, when it should be applied, and what outcomes to expect [10]. Jenkins et al. reviewed practice guidelines dealing with use of spinal x-ray in chiropractic practice and concluded that routine use of x-ray is not in accordance with current evidence and practice guidelines [11]. Hansen et al. reported that chiropractors (at least in Denmark), when surveyed, commonly reported work-related physical acute or overuse injuries in the low back, wrist, thumb and shoulder, but were very satisfied with their job and had a good psychosocial work environment [12]. Gíslason et al., in a survey of chiropractors in Europe, showed that around one fifth identified themselves with an “unorthodox” description of chiropractic care, and that this group were associated with a higher use of x-rays, higher patient visits, absence of differential diagnostic approaches, and less favorable views regarding the benefits of vaccination [13].

Two papers reported on issues relating to education of chiropractors. Innes et al. compared accreditation standards from the Councils of Chiropractic Education between 2010 and 2016 and found that while there had been a move towards a focus on musculoskeletal care, mention of competencies in appraising scientific evidence and evidence-based practice had been removed from the criteria [14]. Funk et al. searched websites of 46 chiropractic educational programs in order to identify and count the mention of the term *subluxation*, and found that it is much more prevalent in descriptions of US-based educational institutions, and in fact the mention of subluxation had increased between 2011 and 2017 [15].

### Papers describing new initiatives and developments in the chiropractic profession

Adams et al. described the aims, core principles, and methodology for the Chiropractic Academy for Research Leadership (CARL). CARL aims to bring together a global network of early career researchers in chiropractic in a global network [16]. Côté et al. described a chiropractic rehabilitation competency framework that was developed by the World Federation of Chiropractic Disability and rehabilitation Committee [17].

## Discussion and debate papers

The final group of papers centered on debates and discussions within chiropractic and their implications for professional unity and legitimacy. Coulter et al. described and discussed the research crisis in research in complementary and alternative medicine, with a focus on chiropractic, and suggested ways to advance this research endeavor [18]. Leboeuf-Yde et al. compared the chiropractic profession with an unhappy marriage between practitioners on a continuum from evidence-based, with a focus on management of musculoskeletal disorders, to conservative and vitalistic, with a focus on addressing subluxations to influence the nervous system. These authors suggested that it may be time to openly discuss separation or divorce so that the two fractions can pursue a separate future [19]. Strahinjevich and Simpson also targeted the schism between the evidence-oriented and the vitalistic groups in chiropractic as seen through the eyes of a first-year student, and discussed both the historic background for this and possible ways forward [20].

## Conclusions

This commentary brings the 2017–2019 series *What is Chiropractic?* to a close. The series will remain open to including future articles, if relevant; this commentary provides a summary of the articles officially included in the series that have been published to date. When we made this call for papers in forming the thematic series, we were optimistic that we would energize the researchers in the profession to answer this question. The 18 papers have contributed significantly to a better understanding of what chiropractic is, where chiropractors practice and function, who seeks their care, what chiropractors do, and how they interact with other healthcare professionals. The series has not contributed with new data on effectiveness of the interventions chiropractors routinely apply to their patients; however, a casual search in PubMed using the search terms *chiropractic* or *chiropractor* for 2018 and 2019 identified over 700 papers, so clearly many scientific papers pertaining to chiropractic have been published in this and other journals alongside this series. Chiropractic and Manual Therapies is an open access journal, and papers in the series have been read widely with over 63,000 downloads (range 978 [18] – 15,000 [19]) and significant activity on social media.

Papers in this series have again revealed deeply rooted disagreements within the chiropractic profession about what chiropractic is, and what it should be, as a profession [13, 19, 20], as well as disagreements and variation in relation to education of chiropractors [14, 15] and chiropractic clinical practice [11]. In our opinion, it is ironic that while chiropractic has a strong presence in large parts of the world [3], is taking on increasingly important roles in disability prevention [6, 7, 17], in the military [5] and in interprofessional care [8] as well as growing research capacity [16], discussions about fundamental values and direction of the profession are unresolved. This unresolved issue creates confusion for stakeholders and threatens to impede professionalization and cultural authority. If chiropractors are to remain relevant in today’s evidence-based healthcare environment, there is an urgent need to agree on, and further describe, what chiropractic is, what chiropractors do and importantly to provide evidence for value of these activities to patients and societies.

## Data Availability

Not applicable.
